# Proposal for improving the education and licensing examination for medical record administrators in Korea

**DOI:** 10.3352/jeehp.2018.15.16

**Published:** 2018-07-11

**Authors:** Hyunchun Park, Hyunkyung Lee, Yookyung Boo

**Affiliations:** 1 Gyeonggi Public Health Policy Institute, Seongnam, Korea; 2 Health Information Research Institute, Korean Medical Record Association, Seoul, Korea; 3 Department of Healthcare Management, College of Health Industry, Eulji University, Seongnam, Korea; Hallym University, Korea

The Korean licensing examination for medical record administrators (MRAs) was introduced in 1985, and as of 2017, approximately 25,000 MRAs have been produced [1]. Over the past 30 years, MRAs have contributed to establishing a foundation for medical record management and building a safe medical environment. Health information management professionals, including MRAs are responsible for improving the quality of healthcare, which they accomplish by planning, collecting, aggregating, analyzing, and disseminating individual patient and aggregate clinical data [2]. Adopting healthcare technology offers the possibility to improve the delivery of care in ways that can provide exceptional quality, promptness, performance, and availability [3]. The greater availability of accurate, complete, and relevant clinical data allows care providers to deliver higher quality, more efficient, and cost-effective care [4]. There is a need to confirm the job competencies of MRAs who are working in an information-oriented environment.

A study was conducted in 2016 to identify the political tasks necessary to improve the education and licensing examination to ensure that MRAs are highly qualified. The present study was performed using the second set of data from a survey and set of focus group interviews carried out in 2016 for a study funded by the Health Personnel Licensing Examination Board fund, entitled “A study on educating and producing excellent MRAs. The present study was exempt from Institutional Review Board approval at the researcher’s institution (IRB approval no., EUIRB 2018-29). Two rounds of interviews targeting 17 professional MRAs, consisting of professors and heads of health information management departments, were conducted. Through discussion and consensus with researchers, improvement tasks were classified into 4 major areas with 9 subsections, as shown in Table 1.

Next, to prioritize the improvement tasks, a survey targeting MRAs was conducted using a questionnaire. The questionnaires were distributed to the 340 MRAs who participated in the 2016 fall conference of the Korean Medical Record Association. Tables presenting the results of this survey can be found in the supplement file (Supplement 1).

The first task that was identified was to change the name of the occupation to strengthen its identity. Most of the MRAs surveyed wanted the name to be changed to ‘medical information manager,’ which would reflect changes in the working environment. Changing the job title is necessary to clearly convey the current role of MRAs in securing complete, accurate, and trustworthy medical information during the life cycle of information. The role of MRAs in foreign countries long ago changed from the management of medical records to the management of health information [5]. The request of MRAs for a change in their job title was accepted by the Korean government, and the related law was reformed at the end of 2017 [6]. The chosen title of ‘health information management specialist’ will be used beginning in December 2018.

The second improvement task was the development of a diversified career path. With advances in information technology (IT), the scope of medical record information management has expanded to include healthcare support and research assistance, health information exchange, medical quality assurance, and providing the basic infrastructure of the medical industry. To develop the groundwork for developing a diversified career path, respondents were surveyed about their desired career path, with the following results in order from most to least preferred: public policy and research, medical information management, and information processing and data analysis. As shown in career guidance programs in the United States [7] and Canada [8], as the importance and value of medical record information is recognized, and the utilization thereof is increasing, the employment of health information management specialists is increasing in various areas, including government and public institutions; health informatics, with the main job of data processing and analysis; and IT infrastructure involved with developing software, applications, systems, and platforms utilizing patient data. Until now, MRAs have primarily been employed at medical institutions. Additionally, previous job analysis and job descriptions of MRA were limited to careers at medical institutions [9]. This might have been the factor that prevented career diversification. Therefore, based on the requests of the MRAs who wanted a greater variety of careers, preparation of educational training programs and corresponding policy developments are needed.

The highest-rated improvement tasks for college educational curricula were in-depth education in major subjects and practice-oriented education. Specifically, the need for education in basic clinical knowledge and in-depth education about medical record information management appeared to be high. Enhancing the job competencies of MRAs and introducing a professional certification system were suggested. Statistics and data processing, disease classification and cancer registration, as well as security and personal information protection appeared to be the most important competencies. Furthermore, the competency to analyze big data and to use artificial intelligence is needed, both to strengthen basic work ability and to increase the value of medical records. Therefore, proper education on health information management, health informatics, and health IT to strengthen these competencies should be prepared. In case of the United States, as the tasks of health information managers have been transformed and expanded in an information-oriented environment, it has been recognized that the existing curriculum cannot fully cover the education of health information management professionals [10]. Therefore, they are redoubling efforts to train highly-qualified, high-level manpower through eliminating 2-year programs, shifting to 4-year degree programs, and establishing master’s degree courses in the field of health information management and health informatics. The convergent evolution of health information management, health informatics, and health IT to strengthen these competencies should be prepared. In an information-oriented environment, under the influence of the fourth industrial revolution, it was confirmed that alicensing examination was expected to serve as a mechanism to verify MRAs’ job competencies.

The opinions on reforming the licensing examination system included support for adding a practical examination on statistical analysis, theoretical subjects based on changes in the work environment, and a practical examination on data processing. Fewer requests for improvement seem to have been made for health information management than for most college educational curricula. The reason for this may be that the MRAs who already hada license and worked in the industry generally did not perceive that the licensing examination needed to be improved in order to strengthen competencies and career path development. The fundamental purpose of the licensing examination is to evaluate an individual’s basic capacity to perform occupational tasks in the field. It is also necessary to verify professionals’ competencies, in order to increase specialization in each industry. However, many experts in the field have pointed out that a considerable training period is needed after recruitment for a new MRA to be able to function autonomously. Therefore, further improvement tasks should be discovered through additional in-depth studies regarding subjects, examination areas, and methods of the licensing examination. Furthermore, it is of the utmost importance to develop questions with high validity and reliability, to strengthen the framework of practical testing, and to introduce a data processing test for work in an information-oriented environment.

We found that the political tasks identified by this study were all closely connected. The first task, changing the job title, has already been completed, and the official title will be changed from ‘medical record administrator’ to ‘health information manager’ beginning in December 2018. The next task is to develop the career path more aggressively to reflect its new name. More intensive research into the job competencies needed in new career fields should be carried out. Based on the findings of such research, the educational curriculum and licensing examination system should be rearranged. Improving the quality of the licensing examination system is the final and most important task needed to create highly qualified MRAs. In an information-oriented environment, under the influence of the fourth industrial revolution, the licensing examination is expected to function as a mechanism to verify MRAs’ job competencies. Furthermore, a detailed action plan for each topic, for each stakeholder who trains MRAs, is needed. These improvements will be the basis for advancing the country’s professional training system, which will produce professionals who are ready for a changing industrial environment.

## Figures and Tables

**Table 1.** Focus group interview results regarding improvement tasks or training highly qualified MRAs

Areas	Improvement tasks
Enhancement of professional identity	Change the title of MRAs
	Develop a career path that reflects diversity
Strengthening of job competencies	Strengthen competencies needed for future environments
	Develop a professional certification system
Improvement of college educational curricula	Unite the names of academic subjects to inspire identity
	Introduce an accreditation system for educational courses
	Improve educational content and environment
Reformation of the licensing examination system	Include exam subjects and contents based on changes in the social environment
	Change to a computer-based testing method

MRA, medical record administrators.

## Supplementary material

Supplement 1. Data files are available from https://doi.org/10.7910/DVN/S5NTZJ
